# Bibliography

**Published:** 1895-10

**Authors:** 


### Bibliography.

General Surgery and Pathology for Dentists. By Edmund W. Roughton, B.S., M.D. (London), F.R.C.S. (England), Warden of the College of St. Mary's Hospital; Assistant Surgeon and Surgeon-in-charge of the Throat and Ear Department, Royal Free Hospital; Honorary Visiting Surgeon National Dental Hospital, etc. With numerous illustrations. Published by J. B. Legg & Co., 289 and 291 Regent St., London, England; S. S. White Dental Manufacturing Company, Philadelphia.

This book of one hundred and thirty-four pages is intended "to supply the student of dentistry with an account of general surgery and pathology sufficiently comprehensive to enable him to practise his profession intelligently, yet concise enough to be easily mastered." While this statement, quoted from the preface, is correct, this book differs from many others prepared to make acquisition of knowledge easy for the student, in that it gives very clear descriptions of pathological subjects and leaves little to be desired in the way of definition.

The book opens, very properly in our judgment, with the subject of inflammation. This is treated very clearly and quite satisfactorily as to detail, giving the generally accepted ideas of the phenomena accompanying it. This is followed by "Bacteria in Relation to Disease, Wounds, Surgical Fever, Shock, Delirium, Hæmorrhage, Fracture, Syphilis," etc., giving a general survey of important matters necessary for the student to acquire in order to have an intelligent conception of general pathology in its intimate relations with the special, with which he is brought daily in contact.

The book is a timely one, being much needed by teachers of this branch to place in the hands of students and thus avoid the large works which are, as a rule, confusing by over-description. If this book should reach a second edition, we would suggest collaboration with a competent dentist, that special matters might be elaborated to the advantage of the student and increase the value of the book without materially enlarging it. The author proposes to meet this want, "The Special Surgery of the Mouth," in another volume. This, to our conception, would be a serious mistake, greatly destroying its value as a text-book.

